# Protective Mechanism of Berberine on Human Retinal Pigment Epithelial Cells against Apoptosis Induced by Hydrogen Peroxide via the Stimulation of Autophagy

**DOI:** 10.1155/2021/7654143

**Published:** 2021-08-13

**Authors:** Shuai Li, Yizhou Jiang, Xingan Xing, Ruohong Lin, Qin Li, Wenshu Zhou, Wei Qiu, Wenhua Zheng

**Affiliations:** ^1^Center of Reproduction, Development & Aging, Faculty of Health Sciences, University of Macau and Institute of Translation Medicine, Faculty of Health Sciences, University of Macau, Taipa, Macau SAR, China; ^2^Hangzhou Medical College, Hangzhou, China; ^3^Neurology Department, 3rd Affiliated Hospital, Sun Yat-sen University, China

## Abstract

Age-related macular degeneration (AMD) is a major cause of severe and irreversible vision loss with limited effective therapies. Diminished autophagy and increased oxidative damage caused by ROS in the retinal pigment epithelium (RPE) have been implicated in the pathogenesis of AMD, and strategies aimed at enhancing autophagy are likely to protect these cells from oxidative damage. We have previously shown that berberine (BBR), an isoquinoline alkaloid isolated from Chinese herbs, was able to protect human RPE cells from H_2_O_2_-induced oxidative damage through AMPK activation. However, the precise mechanisms behind this protective effect remain unclear. Given the essential role of AMPK in autophagy activation, we postulated that BBR may confer protection against H_2_O_2_-induced oxidative damage by stimulating AMPK-dependent autophagy. Our results showed that BBR was able to induce autophagy in D407 cells, whereas autophagy inhibitor PIKIII or silencing of LC3B blocked the protective effect of BBR. Further analysis showed that BBR activated the AMPK/mTOR/ULK1 signaling pathways and that both pharmacological and genetic inhibitions of the AMPK pathway abolished the autophagy-stimulating effect of BBR. Similar results were obtained in primary cultured human RPE cells. Taken together, these results demonstrate that BBR is able to stimulate autophagy in D407 cells via the activation of AMPK pathway and that its protective effect against H_2_O_2_-induced oxidative damage relies on its autophagy-modulatory effect. Our findings also provide evidence to support the potential application of BBR in preventing and treating AMD.

## 1. Introduction

Age-related macular degeneration (AMD) is a leading cause of vision loss in aged people with a great impact in their quality of life. As the worldwide population is growing older, more people are expected to suffer from this serious eye disease in the coming years. Affecting the macula of the retina, it causes a chronic and progressive vision loss [1]. Late AMD can be divided into neovascular (wet) and nonneovascular (dry) forms [1]. In neovascular AMD, choroidal neovascularization causes fluid and blood leakage and leads to macula damage [2]. Nonneovascular AMD, also called geographic atrophy, accounts for approximately 90% of AMD cases and is characterized by the progressive atrophy of retinal photoreceptors, choriocapillaris, and the retinal pigment epithelium (RPE) [3]. The use of anti-VEGF therapies, such as ranibizumab and bevacizumab, has been effective in the treatment of neovascular AMD [4]. However, there is no effective treatment for nonneovascular AMD.

The RPE is a pigmented monolayer which plays a key role in maintaining the function of photoreceptors including nutrient transport, phagocytosis of photoreceptor outer segments, and regeneration of visual pigments [5–9]. The degeneration of RPE has been considered an initial event of AMD [10]. The RPE deteriorates with advancing age and eventually leads to a secondary degradation of photoreceptors and vision loss [3, 10]. Retina is a high oxygen-consuming tissue and produces high levels of ROS by mitochondria respiration, phagocytosis of photoreceptors in RPE, and light exposure [6, 11, 12]. Thus, the balance between ROS generation and clearance is particularly important for the normal function of RPE [11]. However, antioxidant capacity decreases with the increase of age and oxidative damage caused by excess ROS has long been recognized as a major cause of RPE degeneration and AMD [6].

Autophagy is a protective mechanism designed for the degradation of cellular components including those damaged by ROS [6]. Dysregulated autophagy has been implicated in a variety of diseases. Upon the induction of autophagy, autophagosomes engulf cytoplasmic components and degrade them by fusing with the lysosomes [13]. In AMD, the accumulation of dysfunctional mitochondria and toxic proteins due to impaired autophagy could ultimately lead to RPE cell damage or apoptosis [8, 14]. Therefore, strategies aimed at enhancing autophagy may be helpful to protect RPE cells from oxidative damage and prevent or reverse the progression of AMD.

Berberine (BBR) is an isoquinoline alkaloid isolated from Chinese herbs like *Berberis aristate*, *Berberis petiolaris*, *Coptis chinensis*, *Hydrastis canadensis*, and *Caulis mahiniae* [15–17]. A growing body of studies reports that BBR has many pharmacological effects including anti-inflammation, antioxidation, antidiarrheal, antipyretic, and antimicrobial [16, 18]. Besides these activities, recent studies also showed that BBR modulates autophagy by affecting autophagy-related pathways [17]. We have previously reported that BBR protects D407 cells from H_2_O_2_-induced oxidative damage via the AMPK pathway [19]. Given the fact that AMPK is a key modulator of autophagy, we postulated that the protective effect of BBR may be associated with autophagy-modulating activity. In the present study, we aimed to test the hypothesis that BBR stimulates autophagy by activating AMPK, conferring protection against H_2_O_2_-induced oxidative damage.

## 2. Materials and Methods

### 2.1. Materials

Human retinal pigment epithelial cell line D407 was obtained from the cell bank, Sun Yat-sen University (Guangzhou, China). Primary cultured human retinal pigment epithelial cells (hRPE) were obtained from the State Key Laboratory of Ophthalmology, Zhongshan Ophthalmic Center with approval of the Ethics Committee of Zhongshan Ophthalmic Center (2018KYPJ082, 15 May, 2018). 3-(4,5-Dimethylthiazol-2-yl)-2,5-diphenyl tetrazolium bromide (MTT), fetal bovine serum (FBS), bovine serum albumin (BSA), Dulbecco's modified Eagle's medium (DMEM), and 0.25% trypsin were obtained from GIBCOTM (Grand Island, NY, USA). Penicillin/Streptomycin, Lipofectamine 2000 reagent, and DMSO were obtained from Sigma Aldrich (St. Louis, MO, USA). Inhibitors 3-MA and PIKIII were obtained from SelleckChem. Pierce BCA protein assay kit and Halt™ Protease and phosphatase inhibitor cocktail were purchased from Thermo Scientific (Rockford, IL, USA). TUNEL kit and DAPI were obtained from Beyotime Institute of Biotechnology, Shanghai, China. Annexin V-FITC/PI apoptosis detection kit was obtained from BD Biosciences (San Diego, CA, USA). Anti-LC3B, anti-phospho-AMPK, anti-AMPK, anti-phospho-ULK1, anti-ULK1, anti-phospho-mTOR, anti-mTOR, anti-phospho-AKT, anti-AKT, anti-phospho-P38, anti-phospho-ERK1/2, and anti-*β*-actin antibodies were purchased from Cell Signaling Technology (Woburn, MA, USA). GAPDH was purchased from Signalway Antibody, Anti-P62 was purchased from Proteintech, Anti-Rabbit IgG HRP-conjugated secondary antibody was purchased from Promega (Madison, WI, USA), and Alexa 488 secondary antibody was obtained from Invitrogen Co. (Guangzhou, China). LC3 double fluorescent lentivirus autophagy flow detection system, shAMPK, shLC3B, and shULK1 were purchased from Shanghai GeneChem Co., Ltd.

### 2.2. Cell Culture

The D407 cell line was maintained in DMEM supplemented with 10% FBS (heat-inactivated at 56°C for 30 min), 100 *μ*g/ml streptomycin, and 100 U/ml penicillin. hRPE cells were maintained in DMEM/F12 culture medium supplemented with 10% FBS and 1% penicillin/streptomycin. Cell cultures were incubated at 37°C with 5% CO_2_ humidified atmosphere. The medium was replaced every 2-3 days, and cells were subcultured using 0.25% trypsin. For D407 cells, passages 3-8 were used in all experiments.

### 2.3. MTT Assay

Cell viability was determined by MTT assay as previously described [19, 20]. Cells were seeded in 96-well plates at a density of 5 × 10^4^ cells/ml. The next day, the cells were exposed to the reagents for 24 h. The cells were then incubated with MTT (0.5 mg/ml) for 3 h. After this period, the medium was replaced with DMSO. The absorbance at 490 nm was measured by Bio-Rad 680 microplate reader (Thermo Fisher, MA, USA). The assay was repeated for at least 3 times.

### 2.4. TUNEL Staining

TUNEL staining was performed as previously described with minor modifications [21].

Briefly, cells were grown on 96-well plates at a density of 1 × 10^5^ cells/ml. After treatment, the cells were fixed in 4% (*v*/*v*) paraformaldehyde in PBS for 30 min on ice. Cells were then washed with PBS followed by 0.3% Triton X-PBS for 5 min and incubated with TUNEL detection solution at 37° C in the dark for 60 minutes. After this period, the cells were washed 3 times with PBS and observed under a fluorescence microscope at an excitation wavelength ranging between 450 and 500 nm and an emission wavelength ranging between 515 and 565 nm.

### 2.5. Flow Cytometry

Flow cytometry was used to assess cell apoptosis as previously described [22]. Briefly, cells were harvested and washed twice with cold PBS. After washing, cells were resuspended in a binding buffer at a concentration of 1 × 10^6^ cells/ml and then loaded into a flow cytometric tube. Cells were stained with Annexin V-FITC (10 *μ*l) and propidium iodide (PI) (5 *μ*l) for 15 minutes in the dark. Flow cytometry assay was performed using a FACS instrument (BD AccuriC6, BD, USA).

### 2.6. Immunofluorescence of LC3B

Immunofluorescence was performed as previously described [23]. Briefly, after incubation with proteinase K antigen retrieval solution for 15 min followed by 3% H_2_O_2_ for 30 min, the slides were rinsed with PBS and incubated with the primary antibodies (LC3B, 1 : 200) overnight at 4°C. For negative controls, the primary antibody was replaced by nonimmunized serum. The following day, the slides were rinsed and incubated with the corresponding secondary antibody (Alexa Fluor® 488 anti-rabbit IgG) for 1 h followed by three washes in PBS for 15 min. Nuclei were counterstained with DAPI, and images were acquired with a Nikon A1 confocal microscope.

### 2.7. Measurement of Autophagic Flux

The LC3 double fluorescent lentivirus autophagy flow detection system was used for stably and dynamically monitoring changes in cell autophagic flux with the vector hU6-MCS-Ubiquitin-stubRFP-senseGFP-LC3-IRES-puromycin. One overnight after seeding the cells, the medium was aspirated, and the virus solution was added. After 12 h, the virus solution was replaced with normal medium, and the cell cultures were maintained for further 72 h. Drug treatments were performed after infection, and the fluorescence intensity was monitored using a Nikon A1 confocal microscope. Autophagolysosomes and autophagosomes displayed green and red signals, respectively.

### 2.8. Western Blotting

Western blotting was performed as previously described [23, 24]. Briefly, cells were harvested and lysed in a RIPA buffer. Protein concentration was determined using a BCA protein assay kit, and equal amounts of proteins were separated by SDS-PAGE and then transferred to a PVDF membrane. After blocking the membrane with 5% nonfat milk, appropriate antibodies were used to probe the proteins (Anti-LC3B Cat. no. 2639 Rabbit 1 : 1000, anti-P62 Cat. no. 18420-1-AP Rabbit 1 : 1000, anti-phospho-AMPK Cat. no. 2535 s Rabbit 1 : 1000, anti-AMPK Cat. no. 2603 s Rabbit 1 : 1000, anti-phospho-ULK1 Cat. no. 6888 s Rabbit 1 : 1000, anti-ULK1 Cat. no. 8054 Rabbit 1 : 1000, anti-phospho-mTOR Cat. no. 5536 Rabbit 1 : 1000, anti-mTOR Cat. no. 2983 Rabbit 1 : 1000, anti-phospho-AKT Cat. no. 9271 Rabbit 1 : 1000, anti-AKT Cat. no. 4691 Rabbit 1 : 1000, and anti-*β*-actin Cat. no. 12620 s Rabbit). The next day, the membranes were washed 3 times with TBST followed by incubating with secondary antibody for another 2 h. Then, the immunoblotting was performed using an ECL detection kit reagent (BIO-RAD). The intensity of the bands was quantified using ImageJ software.

### 2.9. LC3B, AMPK, and ULK1 Silencing by shRNA

Gene silencing was performed as previously described with minor modifications [19]. Briefly, specifically synthesized siRNA or scrambled siRNA was incubated with Lipo2000 and opti-MEM for 15 mins at room temperature. After incubation, the transfection complexes were added dropwise to the cell cultures. The cells were then maintained in a 37°C incubator for 6 h, and the medium was replaced with complete medium. 48 h after transfection, cells were collected for protein expression analyses or MTT assays.

### 2.10. Statistical Analysis

All experiments were performed in triplicates. The data significance was conducted by GraphPad Prism 7.0 statistical software (GraphPad software, Inc., San Diego, CA, USA). All values were presented as mean ± SEM. Statistical significance among various groups was calculated by one-way ANOVA using post hoc multiple comparisons, when *p* < 0.05 was considered statistically significant.

## 3. Results

### 3.1. BBR Protected D407 Cells from H_2_O_2_-Induced Cell Apoptosis

**Figure 1:**
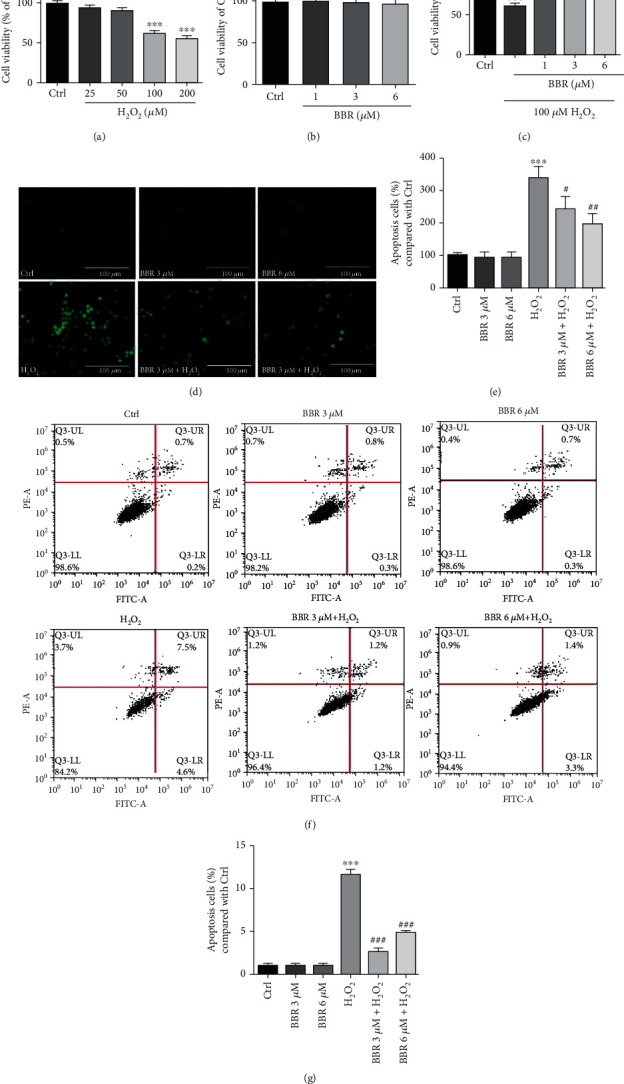
BBR protects D407 cells from H_2_O_2_-induced cell apoptosis. (a) D407 cells were treated with different concentrations of H_2_O_2_ or 0.1% dimethyl sulfoxide (DMSO) (vehicle control) for 24 h, and cell viability was measured by MTT assay. (b) D407 cells were treated with different concentrations of BBR or 0.1% dimethyl sulfoxide (DMSO) (vehicle control) for 24 h, and cell viability was measured by MTT assay (c) D407 cells were pretreated with BBR at indicated concentrations (1 to 6 *μ*M) or 0.1% DMSO (vehicle control) for 2 h and then incubated with or without 100 *μ*M H_2_O_2_ for further 24 h. Cell viability were measured by MTT assay. (d, e) After pretreatment with 3 *μ*M and 6 *μ*M BBR or 0.1% DMSO (vehicle control) for 2 h, D407 cells were incubated with or without 100 *μ*M H_2_O_2_ for another 24 h. Apoptotic cells were observed by TUNEL staining (scale bar = 100 *μ*m). (f, g) After pretreatment with 3 *μ*M and 6 *μ*M BBR or 0.1% DMSO (vehicle control) for 2 h, D407 cells were incubated with or without 100 *μ*M H_2_O_2_ for another 24 h. Apoptotic cells were observed by flow cytometry of PI-Annexin-FITC. The assay was repeated for at least 3 times. ^∗∗∗^*p* < 0.001 versus the control group; ^#^*p* < 0.05, ^##^*p* < 0.01, and ^###^*p* < 0.001 versus the H_2_O_2_-treated group were considered significantly different.

To evaluate H_2_O_2_ cytotoxicity, D407 cells were treated with different concentrations of H_2_O_2_ ranging between 25 and 200 *μ*M for 24 hours. As shown in Figure 1(a), H_2_O_2_ concentration of 100 *μ*M induced a significant reduction of cell viability and was chosen for the following experiments. To evaluate the protective effect of BBR against H_2_O_2_-induced cell death, D407 cells were pretreated with different doses of BBR for 2 h before being exposed to 100 *μ*M H_2_O_2_ for 24 h taking into consideration the doses used previously [19]. As shown in Figure 1(c), 1, 3, and 6 *μ*M BBR pretreatment significantly attenuated H_2_O_2_-induced cell viability loss. Importantly, incubation of cells with BBR alone for 24 hours at concentrations ranging from 1 to 6 *μ*M did not cause any cytotoxicity (Figure 1(b)). Further assessment of the protective effects of BBR revealed that pretreatment of cells with 3 and 6 *μ*M of BBR resulted in a significant reduction of H_2_O_2_-triggered apoptotic cell death (Figures 1(d) and 1(e)). These findings were confirmed by flow cytometry (Figures 1(e) and 1(g)). BBR treatment alone did not have any obvious cytotoxic effect (Figures 1(d)–1(g)).

### 3.2. BBR Stimulates Autophagy in D407 Cells

**Figure 2:**
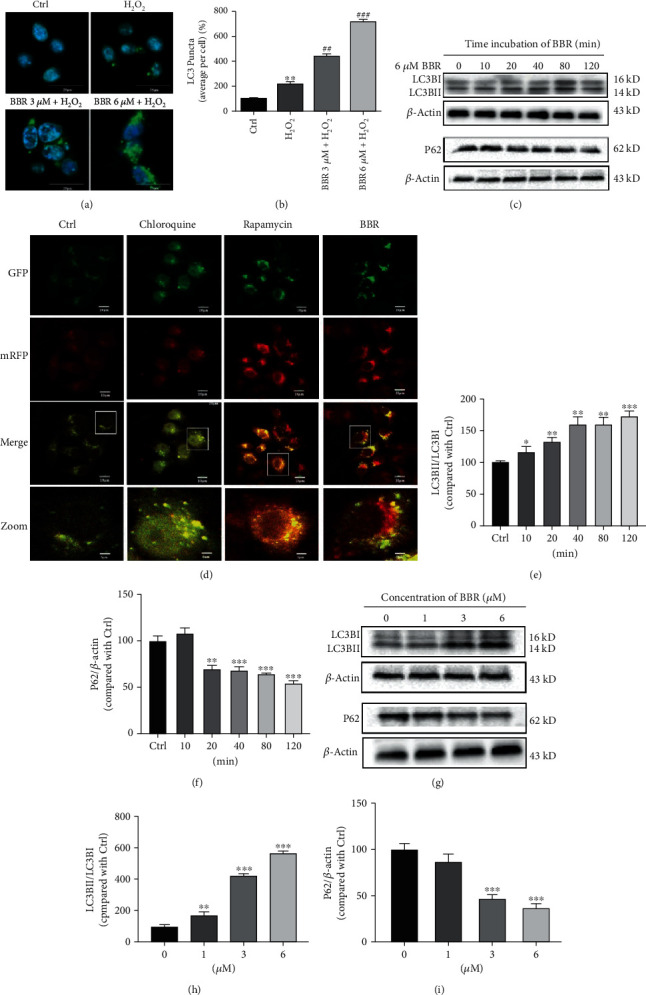
BBR stimulates autophagy in D407 cells. (a) Confocal microscopy examination of LC3B expression. (b) Quantification of LC3B puncta. (c) D407 cells were treated with 6 *μ*M BBR for different time periods as indicated, and LC3B, P62, and *β*-actin were detected by western blotting with specific antibodies. (d) Autophagic flux was detected by LC3 double fluorescent lentivirus autophagy flow detection system; a tandem GFP-RFP-LC3 fusion protein was treated with chloroquine (CQ, 60 *μ*M), rapamycin (1 *μ*M), and berberine (BBR, 6 *μ*M) for 2 h. Confocal microscopy was used to examine the autophagic flux (scale bar = 10 *μ*m). (e, f) Quantification of the representative protein bands from western blotting. (g) D407 cells were treated with various concentrations of BBR for 2 h, and the expression of LC3B, P62, and *β*-actin was detected by western blotting with specific antibodies. (h, i) Quantification of the representative protein bands from western blotting. The assay was repeated for at least 3 times. ^∗^*p* < 0.05, ^∗∗^*p* < 0.01, and ^∗∗∗^*p* < 0.001 versus the control group; ^##^*p* < 0.01, ^###^*p* < 0.001 versus the H_2_O_2_-treated group were considered significantly different.

Autophagy is an important protective mechanism that removes damaged cellular components and protects cells from oxidative damage. Therefore, we postulated that BBR could confer protection against H_2_O_2_-induced oxidative damage by enhancing autophagy. To test this hypothesis, we assessed the effect of BBR on the expression of LC3B, a widely used autophagy marker. Obtained results revealed that H_2_O_2_ induced an increase in the number of LC3B puncta per cell, an effect that was further strengthened by the cotreatment with 3 *μ*M and 6 *μ*M of BBR (Figures 2(a) and 2(b)). These findings indicate that 3 *μ*M and 6 *μ*M of BBR-induced enhancement of autophagy in D407 cells upon H_2_O_2_ treatment is likely to occur in order to confront H_2_O_2_-induced oxidative damage. To confirm this claim, investigation of whether BBR is able to enhance the autophagic flux in D407 cells was performed by detecting the expression of tandem GFP-RFP-LC3 fusion protein using a LC3 double fluorescent lentivirus autophagy flow detection system. As shown in Figure 2(d), treatment with chloroquine (CQ, 60 *μ*M), an agent known to be able to inhibit the fusion of autophagosomes and lysosomes and used as a negative control, resulted in the inhibition of the autophagy flux. Contrarily, rapamycin (1 *μ*M) was able to enhance the autophagic flux and was used as a positive control. Similar to rapamycin, treatment of the cells with 6 *μ*M of BBR increased the autophagic flux. Further study of the autophagy-modulating effect of BBR in D407 cells comprehended the assessment of LC3B and protein P62 (another autophagy marker, which degrades in autophagy) expression levels [25, 26]. Treatment of D407 cells with 6 *μ*M of BBR for 0-120 minutes induced a significant upregulation of the ratio of LC3BII/LC3BI starting at 10 mins and peaking at 120 mins after exposure to BBR and a significant decrease of P62 levels after 20 mins (Figures 2(c), 2(e), and 2(f)). Exposure of D407 cells to varying concentrations (0-6 *μ*M) of BBR for 2 h revealed that treatment with 1, 3, and 6 *μ*M of BBR resulted in the marked increase of LC3B expression and decrease of P62 levels (Figures 2(g)–2(i)). Taken together, BBR treatment upregulated LC3B expression and downregulated P62 levels in a time- and dose-dependent manner, respectively.

### 3.3. Autophagy Inhibitors PIKIII and 3-MA or Silencing of LC3B Blocked the Protective Effect of BBR in D407 Cells

**Figure 3:**
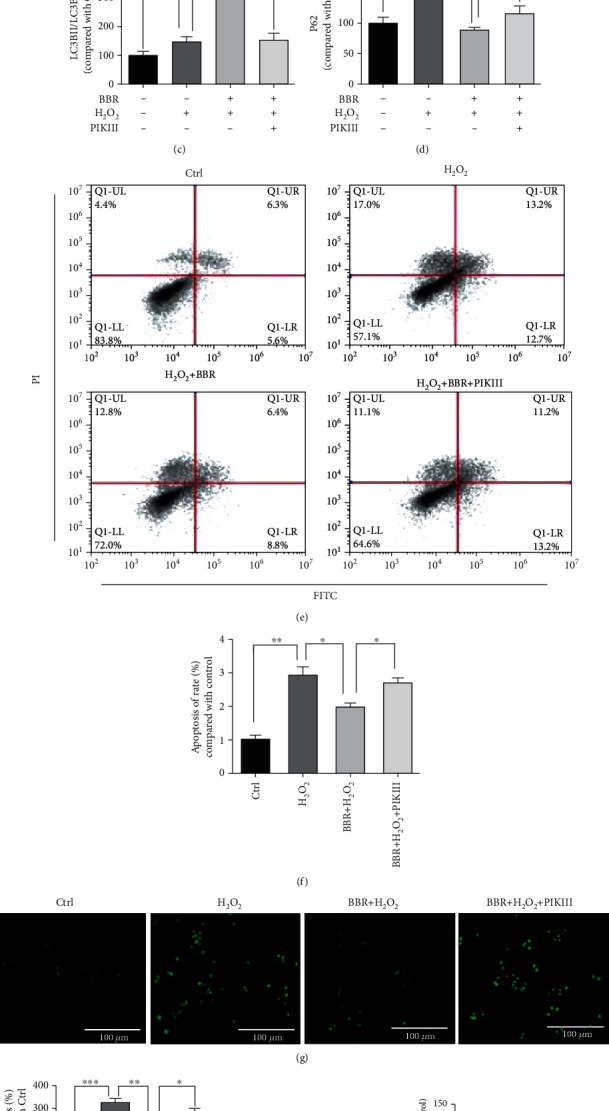
Autophagy inhibitor PIKIII or silencing of LC3B blocked the protective effect of BBR in D407 cells. (a–d) D407 cells were pretreated with 5 *μ*M PIKIII for 2 h and 6 *μ*M BBR for 2 h and then incubated with or without H_2_O_2_ for a further 24 h. Cell viability was measured by MTT assay, and LC3B, P62, and *β*-actin were detected by western blotting with specific antibodies. D407 cells were pre-treated with 5 *μ*M PIKIII for 2 h and 6 *μ*M BBR for 2 h and then incubated with or without H_2_O_2_ for a further 24 h. Apoptotic cells were observed by flow cytometry of PI-Annexin-FITC (e, f) and TUNEL staining (scale bar = 100 *μ*m) (g, h). (i) D407 cells transfected with si-CTRL and/or si-LC3B; the expression of LC3B was detected by western blotting with specific antibodies. (j) D407 cells transfected with si-CTRL and/or si-LC3B; then, cells treated with BBR (6 *μ*M) for 2 h were exposed with or without H_2_O_2_ (100 *μ*M) for 24 h in 96-well plate. Cell viability was measured by MTT assay. The assay was repeated for at least 3 times. ^∗^*p* < 0.05, ^∗∗^*p* < 0.01, and ^∗∗∗^*p* < 0.001 were considered significantly different.

To investigate if the protective effect of BBR against H_2_O_2_-induced cell death was dependent of its ability to enhance autophagy, the autophagy inhibitor PIKIII was added to cells prior BBR treatment. As shown in Figure 3(a), the protective effect of 3 and 6 *μ*M of BBR in D407 cells was abolished by PIKIII. In addition, western blot analysis suggested that PIKIII efficiently blocked the effect of BBR on LC3B and P62 protein levels (Figures 3(b)–3(d)). Results from flow cytometry (Figures 3(e) and 3(f)) and TUNEL staining (Figures 3(g) and 3(h)) also showed that the protective effect of BBR against H_2_O_2_-induced cell apoptosis was blocked in the presence of PIKIII. Further confirmation of these findings was made by using shRNA to knockdown the expression of LC3B. Results of western blot analysis revealed that the knockdown was efficient (Figure 3(i)), and, as expected, 6 *μ*M BBR pretreatment failed to rescue cell viability loss caused by H_2_O_2_ in these conditions (Figure 3(h)). Additionally, the autophagy inhibitor 3-MA was also able to block the protective effect of BBR in D407 cells (Fig. S1). Therefore, enhanced autophagy is essential for the protective effect of BBR against H_2_O_2_-induced cell death.

### 3.4. AMPK/mTOR/ULK1 Signaling Pathway Is Involved in the Protective Effect of BBR in D407 Cells

**Figure 4:**
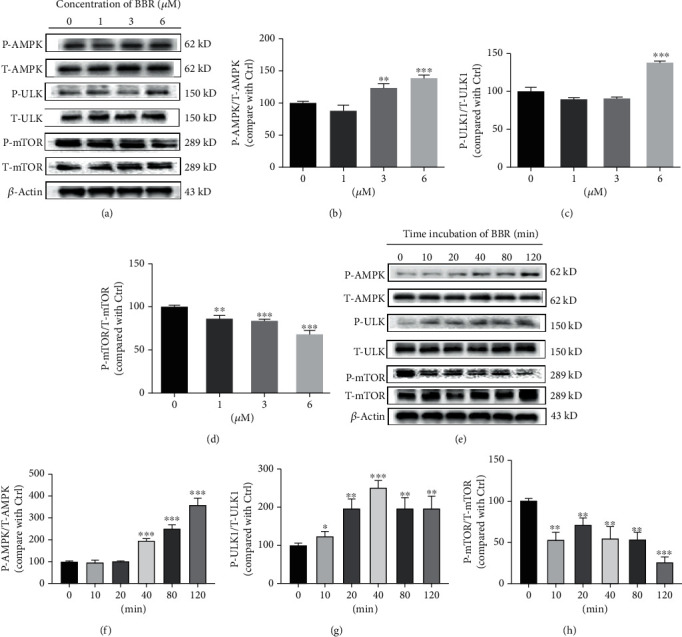
AMPK/mTOR/ULK1 signaling pathway is involved in the protective effect of BBR in D407 cells. (a) D407 cells were treated with variable concentrations of BBR for 120 min, and the expression of phosphorylated AMPK (P-AMPK), total AMPK (T-AMPK), phosphorylated ULK (P-ULK), total ULK (T-ULK), phosphorylated mTOR (P-mTOR), total mTOR (T-mTOR), and *β*-actin was detected by western blotting. (b–d) Quantification of the representative protein bands from western blotting. (e) D407 cells were treated with 6 *μ*M BBR during different time periods as indicated, and the expression of phosphorylated AMPK (P-AMPK), total AMPK (T-AMPK), phosphorylated ULK (P-ULK), total ULK (T-ULK), phosphorylated mTOR (P-mTOR), total mTOR (T-mTOR), and *β*-actin was detected by western blotting. (f–h) Quantification of the representative protein bands from western blotting. The assay was repeated for at least 3 times. ^∗∗^*p* < 0.01, ^∗∗∗^*p* < 0.001 versus the control group were considered significantly different.

**Figure 5:**
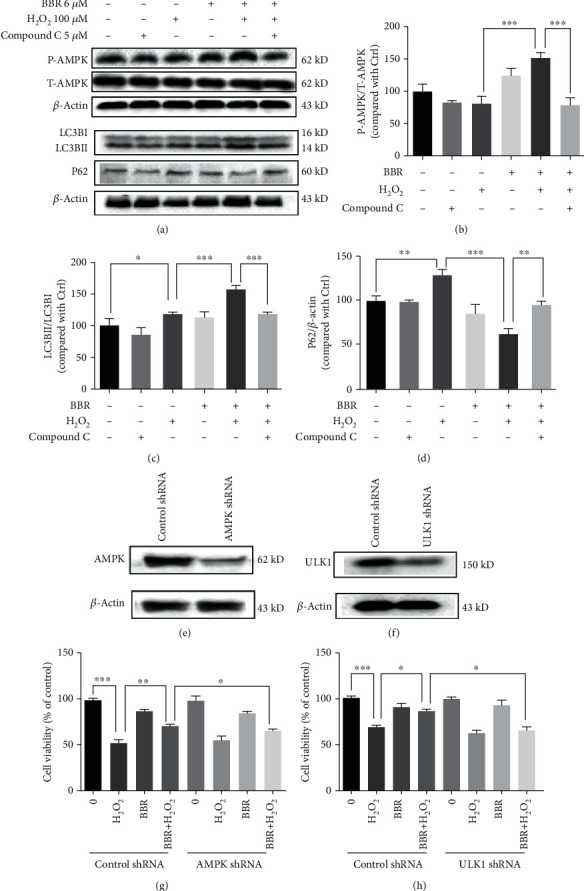
AMPK specific inhibitor compound C blocked the autophagy-stimulating and protective effect of BBR. (a) D407 cells were pretreated with 5 *μ*M compound C for 30 min and 6 *μ*M BBR for 2 h and then incubated with or without H_2_O_2_ for further 2 h. The expression of phosphorylated AMPK, total AMPK, LC3B, P62, and *β*-actin was detected by western blotting. (b–d) Quantification of the representative protein bands from western blotting. (e) Cells were transfected with AMPK shRNA for 48 h, and the expression of AMPK was detected by western blotting. (f) Cells were transfected with AMPK shRNA, treated with 6 *μ*M BBR or 0.1% DMSO (vehicle control) for 2 h, and then incubated with or without 100 *μ*M H_2_O_2_ for 24 h. Cell viability was measured by MTT assay. (g) Cells were transfected with ULK1 shRNA for 48 h, and the expression of ULK1 and *β*-actin was detected by western blotting. (h) Cells were transfected with ULK1 shRNA, treated with 6 *μ*M BBR or 0.1% DMSO (vehicle control) for 2 h, and then incubated with or without 100 *μ*M H_2_O_2_ for 24 h. Cell viability was measured by MTT assay. The assay was repeated for at least 3 times. ^∗^*p* < 0.05, ^∗∗^*p* < 0.01, and ^∗∗∗^*p* < 0.001 were considered significantly different.

The AMPK/mTOR/ULK1 signaling pathway plays a key role in autophagy regulation. AMPK stimulates autophagy by directly activating ULK1 through phosphorylation, whereas mTOR inhibits ULK1 activity by phosphorylating it at another site [27]. In addition, AMPK indirectly activates ULK1 through inhibition of mTORC1. In order to address the molecular mechanisms underlying BBR-mediated autophagy, the total and phosphorylated protein levels of AMPK, mTOR, and ULK1 were tested. As demonstrated in Figures 4(a)–5(d), BBR dose dependently stimulated the phosphorylation of AMPK and ULK1 and inhibited mTOR phosphorylation. Likewise, BBR also stimulated the phosphorylation of AMPK and ULK1 and inhibited mTOR phosphorylation in a time-dependent manner (Figures 4(e)–4(h)). These findings suggest that BBR may regulate autophagy through AMPK signaling.

### 3.5. AMPK Specific Inhibitor Compound C Blocked the Autophagy-Stimulating and Protective Effect of BBR

To investigate whether AMPK is required for BBR-mediated autophagy and protection, we tested the effect of BBR in D407 cells when AMPK signaling is inhibited by compound C. Western blot analysis showed that compound C blocked BBR-induced AMPK activation, elevated LC3B, and decreased P62 levels (Figures 5(a)–5(d)), indicating that BBR stimulates autophagy through the AMPK pathway. To further study the role of AMPK and autophagy in BBR-mediated protective effects, we silenced AMPK by shRNA, and the knockdown efficiency is shown in Figure 5(e). The results of the MTT assay demonstrate that BBR failed to protect D407 cells from H_2_O_2_-induced cell viability loss when AMPK was knocked down (Figure 5(g)). Further knockdown of ULK1 also abolished the protective effect of BBR (Figures 5(f) and 5(h)). Taken together, these data suggest that BBR confers protection against H_2_O_2_-induced oxidative damage by stimulating AMPK-dependent autophagy.

### 3.6. BBR Protected Primary Cultured Human RPE Cells against H_2_O_2_-Induced Injury

**Figure 6:**
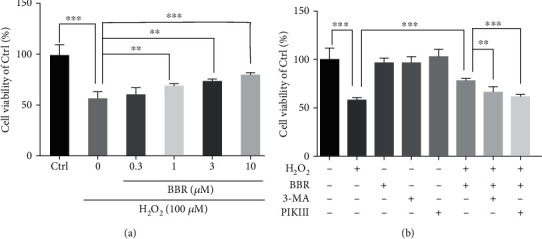
BBR protected primary cultured human RPE cells against H_2_O_2_ induced injury. (a) Primary cultured hRPE cells were pretreated with different concentrations of BBR (0.3 to 10 *μ*M) for 2 h and then incubated with or without H_2_O_2_ for further 24 h. Cell viability was measured by MTT assay. (b) Primary cultured hRPE cells were pretreated with 5 *μ*M PIKIII or 10 *μ*M 3-MA for 2 h, followed by 10 *μ*M BBR for 2 h and then incubated with or without H_2_O_2_ for further 24 h. Cell viability was measured by MTT assay. The assay was repeated for at least 3 times. ^∗∗^*p* < 0.01, ^∗∗∗^*p* < 0.001 were considered significantly different.

Assessment of the protective effect of BBR in primary cultured human RPE cells revealed a similar protective effect of BBR against H_2_O_2_-induced cell viability loss (Figure 6(a)). Moreover, the use of PIKIII and 3-MA autophagy inhibitors blocked this protective effect (Figure 6(b)), indicating that the BBR protection in hRPE cells occurs through autophagy mediation.

## 4. Discussion

The retina is a high energy-demanding tissue, thus making RPE cells especially susceptible to oxidative damage [11]. As oxidative stress is a recognized risk factor of AMD, recent studies have been focusing on exploring the potential use of antioxidants in treating this disease. For example, several antioxidant vitamins and minerals have been shown to be effective in reducing the risk of progression of the disease and loss of visual acuity [28].

Additionally, it was recently reported that dietary plant-derived antioxidants such as anthocyanins were able to protect RPE cells from light-induced oxidative damage [29].

BBR has shown antioxidant capacities in many types of cells [30, 31]. In addition, we have previously reported that BBR was able to reverse H_2_O_2_-induced cell viability loss and to restore the abnormal changes in nuclear morphology, intracellular ROS, mitochondrial membrane potential, and caspase activation [19]. However, the molecular mechanisms behind these protective effects were unclear.

Autophagy is an evolutionally conserved cytoprotective mechanism critical for the protection against oxidative damage. The pathogenesis of AMD has been associated with impaired autophagy, and strategies to enhance autophagy have been shown promising effects by being able to protect RPE cells from oxidative damage [32]. For example, the mTOR inhibitor rapamycin is a widely used autophagy activator and showed protective effect against H_2_O_2_-induced oxidative damage in RPE cells [8]. However, a variety of side effects of rapamycin, such as respiratory and urinary infections, glucose intolerance, diabetes, and thrombocytopenia, have been reported [33, 34], emphasizing the importance to develop a new drugs.

BBR has been shown to promote autophagy in different tissues. In the liver, BBR has been reported to be able to protect the livers under cholesterol overloading by ameliorating blocked autophagic flux [35]. In macrophages, a BBR-mediated sonodynamic therapy induced autophagy via the PI3K/AKT/mTOR signaling pathway [36]. Another study found that BBR could activate AMPK and enhance autophagy to reduce high glucose-induced apoptosis of mouse podocytes [37]. However, whether BBR has a regulatory role in autophagy in RPE cells was unknown. Previously, we found that BBR could activate the AMPK pathway in D407 cells [19]. As AMPK plays a key role in autophagy regulation, we hypothesized that BBR may be able to stimulate autophagy in these cells. Initially, we found that BBR induced an increase in the number of LC3B puncta per cell and, by using a double fluorescent lentivirus autophagy flow detection system, we were able to show that BBR could enhance the autophagic flux in D407 cells. Western blot analysis demonstrated that BBR treatment increased LC3B and decreased P62 expression levels. These findings supported our hypothesis that BBR could stimulate autophagy in D407 cells. Interestingly, BBR appears to have a dual effect on autophagy as other studies reported that BBR inhibits autophagy in other cell types. For instance, BBR was shown to inhibit basal autophagy in mouse adipocytes [38]. Also, in H9c2 myocytes, BBR could inhibit the expression of autophagy-related proteins such as SIRT1, BNIP3, and Beclin-1 to suppress ischemia/reperfusion-caused excessive autophagy [39]. Therefore, it seems that the impact of BBR on autophagy is tissue-specific, and the precise mechanisms of BBR-regulated autophagy in difference tissues are an interesting question that needs further investigation.

We then wanted to know whether the protective effects of BBR against H_2_O_2_-induced oxidative damage were attributed to its autophagy modulatory effect. Results of MTT, flow cytometry, and TUNEL assays demonstrated that autophagy inhibition blocked the protective effect of BBR against H_2_O_2_-induced cell viability loss and apoptosis, indicating that autophagy is involved in BBR-mediated protection. As a key regulator of autophagy, AMPK can directly phosphorylate and activate ULK1 to promote autophagy, while mTORC1 phosphorylates ULK1 at a different site to disrupt ULK1-AMPK interaction [27, 40]. In addition, AMPK stimulates autophagy by inhibiting mTOR-mediated suppression of ULK1 [41]. Given that BBR is able to activate AMPK in D407 cells, we checked the effects of BBR on mTOR and ULK1 phosphorylation. As expected, BBR treatment resulted in decreased mTOR phosphorylation and increased ULK1 phosphorylation. Additionally, both pharmacological and genetic inhibition of AMPK and genetic inhibition of ULK1 blocked BBR protective effect. Taken together, our data suggest that BBR protects D407 cells against H_2_O_2_-induced oxidative damage by stimulating autophagy via AMPK activation. However, the autophagy regulatory effect of BBR may be not solely dependent on AMPK activation, as we also found that BBR stimulated AKT phosphorylation (Fig. S2) and inhibited P38 and ERK1/2 phosphorylation (Fig. S3) in D407 cells. Therefore, as a multitarget drug, BBR may also regulate autophagy through the Akt/FoxO3 or Akt/mTOR pathway [42, 43].

Moreover, the ERK/mTOR pathway may also be involved in BBR-induced autophagy. This is consistent with previous reports that BBR can led to high autophagy flux by inhibition of the ERK1/2-dependent mTOR pathway in hepatic steatosis [44].

At last, we proved that BBR has a similar protective effect in primary cultured human RPE cells and demonstrated that BBR could protect RPE cells from H_2_O_2_-induced oxidative damage by enhancing autophagy via AMPK activation. Our findings reveal the molecular mechanisms underlying the antioxidant effects of BBR in RPE cells supporting its potential application as a novel autophagy activator to prevent and treat AMD.

## Data Availability

The data used to support the findings of this study are included within the article.

## Supplementary Materials

Supplementary Materials Figure S1: Autophagy inhibitor 3-MA blocked the protective effect of BBR in D407 cells. Figure S2: BBR stimulated AKT phosphorylation in D407 cells. Figure S3: BBR inhibited ERK1/2 and P38 phosphorylation in D407 cells.
