# Supplementary material for: Protective Mechanism of Berberine on Human Retinal Pigment Epithelial Cells against Apoptosis Induced by Hydrogen Peroxide via the Stimulation of Autophagy
Source: Oxid Med Cell Longev. 2021 Aug 13;2021:7654143. doi: 10.1155/2021/7654143 (PMC8378965; doi:10.1155/2021/7654143)
Supplement: Supplementary Materials — Figure S1: Autophagy inhibitor 3-MA blocked the protective effect of BBR in D407 cells. Figure S2: BBR stimulated AKT phosphorylation in D407 cells. Figure S3: BBR inhibited ERK1/2 and P38 phosphorylation in D407 cells. [file 7654143.f1.docx]

**Supplementary figures**

**Supplementary figure 1**


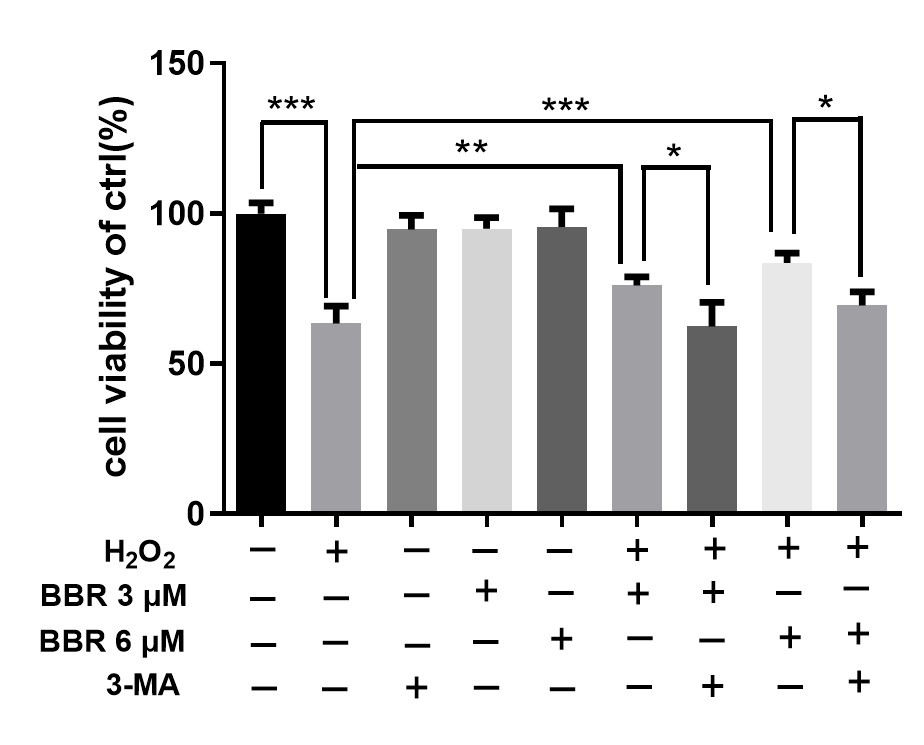


**Fig.S1 Autophagy inhibitor 3-MA blocked the protective effect of BBR in D407 cells.** D407 cells were pre-treated with 10 µM 3-MA for 2 h and 3 µM or 6 µM BBR for 2 h and then incubated with or without H_2_O_2_ for further 24 h. Cell viability was measured by MTT assay. The assay was repeated for at least 3 times. * p < 0.05, ** p<0.01, *** p < 0.001 was considered significantly different.

**Supplementary figure 2**


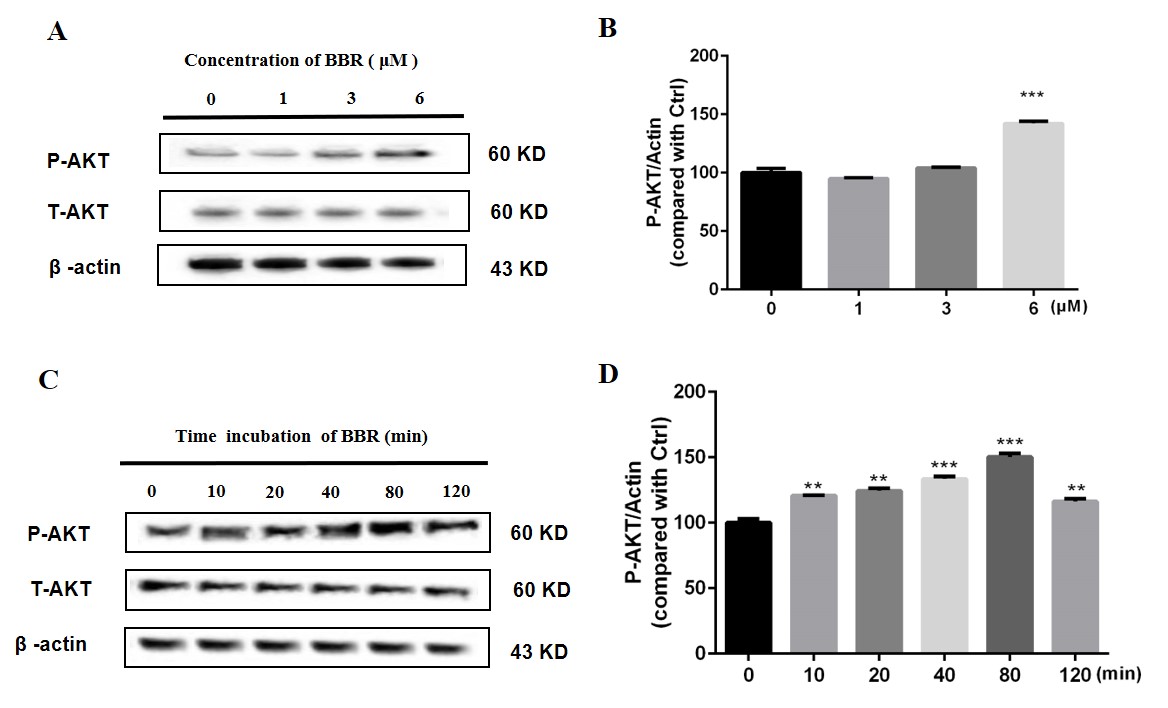


**Fig.S2 BBR stimulated AKT phosphorylation in D407 cells.** (A, B) D407 cells were treated with different concentrations of BBR for 120 min and the expression of phosphorylated AKT (P-AKT, total AKT (T-AKT) and β-actin was assessed by Western blotting. (C) D407 cells were treated with 6 µM BBR for different time periods as indicated in the figure and the expression of phosphorylated AKT (P-AKT), total AKT (T-AKT) and β-actin was detected by Western blotting. (D) Quantification of the representative protein bands from Western blotting. The assay was repeated for at least 3 times. ** p < 0.01, *** p < 0.001, versus the control group was considered significantly different.

**Supplementary figure 3**


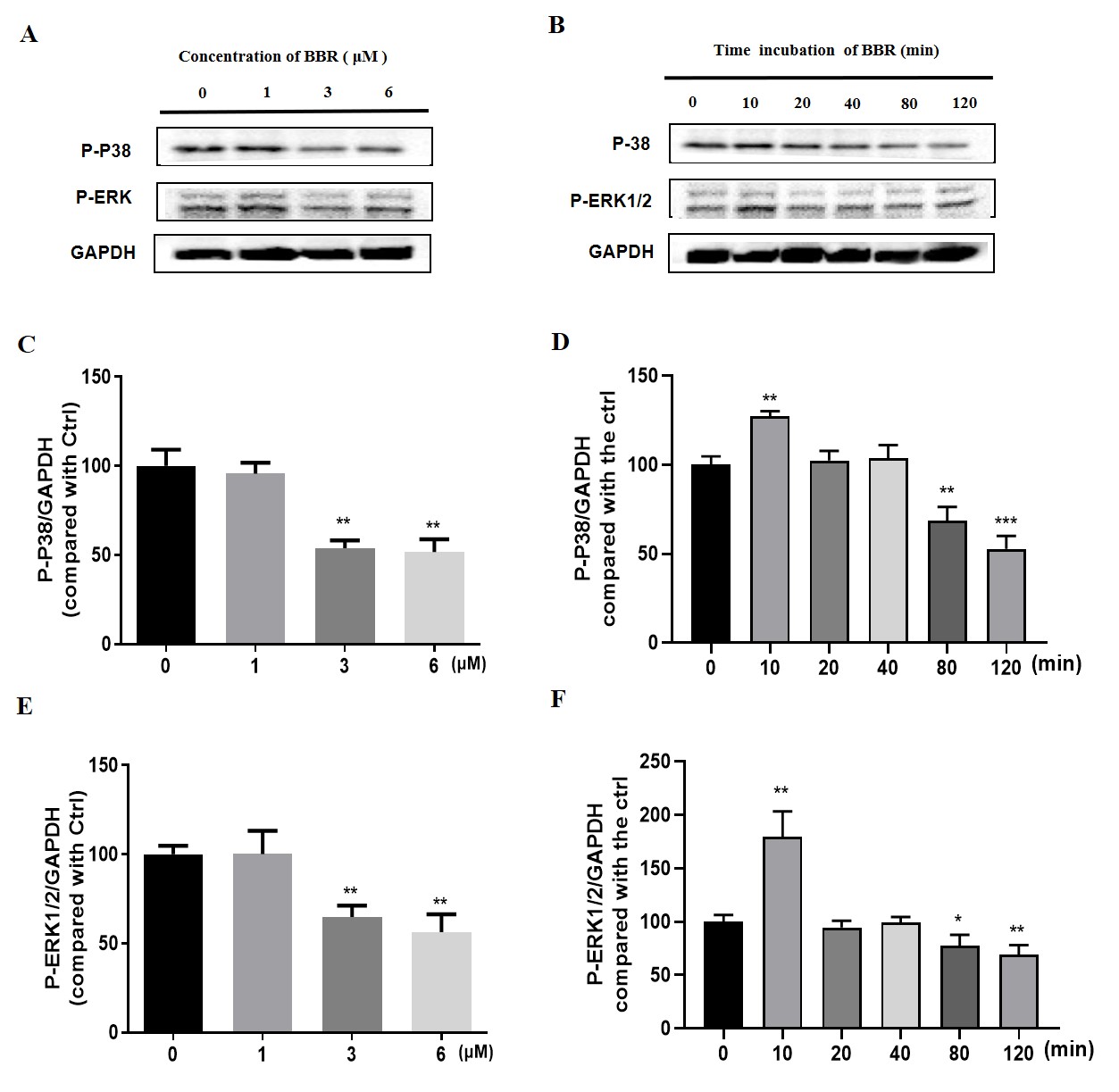


**Fig.S3 BBR inhibited ERK1/2 and P38 phosphorylation in D407 cells.** (A,C,E ) D407 cells were treated with different concentrations of BBR for 120 min and the expression of phosphorylated P-P38,P-ERK1/2 and GAPDH were assessed by western blotting. (B,D,F) D407 cells were treated with 6 µM BBR for different time periods as indicated in the figure and the expression of P-P38,P-ERK1/2 and GAPDH were detected by western blotting. * p < 0.05, ** p < 0.01, *** p < 0.001, versus the control group was considered significantly different.
